# Determinants of Antibiotic Consumption - Development of a Model using Partial Least Squares Regression based on Data from India

**DOI:** 10.1038/s41598-018-24883-1

**Published:** 2018-04-23

**Authors:** Ashok J. Tamhankar, Shreyasee S. Karnik, Cecilia Stålsby Lundborg

**Affiliations:** 1 Global Health- Health Systems and Policy: Medicines, focusing antibiotics, Department of Public Health Sciences, Karolinska Institutet, Tomtebodavägen 18A, 171 77 Stockholm, Sweden; 2 Indian Initiative for Management of Antibiotic Resistance, Department of Environmental Medicine, R.D. Gardi Medical College, Ujjain, 456006 India

## Abstract

Antibiotic resistance, a consequence of antibiotic use, is a threat to health, with severe consequences for resource constrained settings. If determinants for human antibiotic use in India, a lower middle income country, with one of the highest antibiotic consumption in the world could be understood, interventions could be developed, having implications for similar settings. Year wise data for India, for potential determinants and antibiotic consumption, was sourced from publicly available databases for the years 2000–2010. Data was analyzed using Partial Least Squares regression and correlation between determinants and antibiotic consumption was evaluated, formulating ‘Predictors’ and ‘Prediction models’. The ‘prediction model’ with the statistically most significant predictors (root mean square errors of prediction for train set-377.0 and test set-297.0) formulated from a combination of Health infrastructure + Surface transport infrastructure (HISTI), predicted antibiotic consumption within 95% confidence interval and estimated an antibiotic consumption of 11.6 standard units/person (14.37 billion standard units totally; standard units = number of doses sold in the country; a dose being a pill, capsule, or ampoule) for India for 2014. The HISTI model may become useful in predicting antibiotic consumption for countries/regions having circumstances and data similar to India, but without resources to measure actual data of antibiotic consumption.

## Introduction

Antibiotic resistance has become a critical threat to health globally, with most severe consequences for people and settings with constrained resources^1,2^. In 2015,the World Health Assembly endorsed a global action plan (GAP) on ‘antimicrobial resistance’, with special emphasis on ‘antibiotic resistance’ and all countries were invited to prepare national action plans by May 2017^3^. The United Nations general assembly passed a resolution in September 2016, whereby tackling antimicrobial resistance has been placed as the topmost agenda for all nations of the world^2^.

Recognized key drivers for development and dissemination of antibiotic resistance are antibiotic use in both human and animal health sectors and the antibiotic residues in the environment^3^. Few countries, except for some high and higher middle income countries have, however, at present the capability of monitoring their antibiotic consumption. India, a lower middle income country with close to one fifth of the total global population, has been reported as a country with one of the highest human antibiotic consumption in the world (2010 data-12.9 billion standard units (standard units = number of doses sold in the country; a dose being a pill, capsule, or ampoule) annually as against 10 billion units in China and 6.8 billion units in the US)^4^. A high antibiotic consumption is likely to result in increased development and dissemination of antibiotic resistance, so if the determinants for human antibiotic use in India could be understood, interventions could be more easily developed to address them.

A range of determinants governing human antibiotic use or health seeking behaviour in various countries/regions, such as social, demographic, economic, climatic, educational, cultural and health care related, have been reported or discussed in literature^5–14^. In case of India, such studies on antibiotics are mostly related to antibiotic use in hospitals or community or focus on specific disease^15–18^. As nationwide studies on factors determining human antibiotic consumption are lacking for India, it was of interest to explore the determinants for antibiotic consumption for India. Further, India being a medium human development index country (human development index-HDI rank 131 amongst188 countries)^19^, it was considered that such a study could also have implications for countries with proximate HDI and similar settings.

## Results

**Figure 1:**
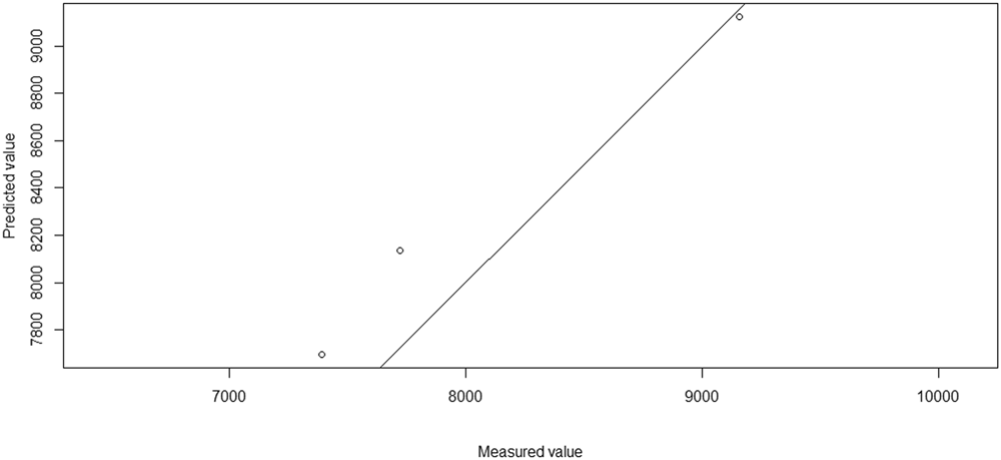
Human antibiotic consumption in India: measured value vs predicted value (open circles) plot for test set for the model with 2 components.

The various primary determinants along with their combinations (summary of primary determinants within categories) termed as derived determinants that were used to build the prediction model for antibiotic consumption in India using the ‘R’ statistical language platform^20^ and partial least squares (PLS) regression^21^ are presented in Table 1. The Root mean square errors for prediction (RMSEP) for the training set using cross-validation for models with varying number of components are presented in Table 2. Figure 1 presents the measured value vs predicted value plot for test set for the model with 2 components. The RMSEP for the 2 component model was lower than for other models (Table 2). Hence, the model with 2 components was considered to give better estimates of antibiotic consumption as compared to other models.

**Table 1:** Human antibiotic consumption in India: Determinants and their Variable importance in projection (VIP) scores.

Name of the determinant	Information Source	Reference/Remark	Determinant abbreviation (in Fig. 1b)	Variable importance in projection- 2 components
*Demographic factors*				
Population (total)	World Bank^50^	Bu, *et al*.^14^	pop	1.69733
Population age over 65 years (% of total)	World Bank^50^	Filippini, *et al*.^10^	pop_65_ov	0.000006
Population density (people per sq. km of land area)	World Bank^50^	Álvarez, *et al*.^12^ Filippini, *et al*.^10^	pop_dens	0.000002
Population, ages 0–14 (% of total)	World Bank^50^	Filippini, *et al*.^10^	pop_0_14	0.0000004
Population, ages 15–64 (% of total)	World Bank^50^	This paper	pop_15_64	0.0000002
*Socioeconomic factors*				
GDP (billions of $) - PPP	World Bank^50^	Bu, *et al*.^14^	gdp_ppp	0.004929
Gross national income GNI per capita ppp (current international $)	World Bank^50^	Filippini, *et al*.^10^	gni	0.001005
GDP (billions of $) - number	World Bank^50^	Bu, *et al*.^14^	gdp_bn	0.000652
Population Below Poverty Line (%)	PC, GoI^51^	Filippini, *et al*.^10^	pop_bpl	0.000029
Health expenditure per capita (US$)	World Bank^50^	This paper	hlth_exp_pc	0.000011
Health expenditure (% of PPP)	World Bank^50^	This paper	hlth_exp_ppp	0.000005
Health expenditure, total (% of GDP)	World Bank^50^	This paper	hlth_exp_tot	0.000003
Life expectancy at birth, total (years)	World Bank^50^	This paper	life_exp_brth	0.0000002
*Health system*				
Health infrastructure (Derived determinant)	MSPI OGD, GoI^52^	This paper	hlth_wf	0.663662
Total number of hospital beds	MSPI OGD, GoI^52^	Bu, *et al*.^14^	tot_beds	0.308826
Total number of providers	MSPI OGD, GoI^52^	This paper	providers	0.126669
Total number of professionals and providers	MSPI OGD, GoI^52^	García-Rey, *et al*.^8^	profes_provider	0.098591
Total number of professionals	MSPI OGD, GoI^52^	García-Rey, *et al*.^8^	profes	0.062381
Total number of hospitals	MSPI OGD, GoI^52^	Bu, *et al*.^14^	tot_hosp	0.003175
*Healthcare*				
Child vaccination rates Diphtheria, tetanus, pertussis, % of children	Organisation for Economic Co-operation and Development^53^	This paper	dpt_vac_per	0.000013
Child vaccination rates Measles, % of children	Organisation for Economic Co-operation and Development^53^	This paper	meas_vac_per	0.000004
Infant mortality rate (per 1,000 live births)	World Bank^50^	This paper	inf_mor	0.000003
*Disease specific figures*				
Infectious disease cases (Derived determinant)	World Health Organisation Global Health Observatory^54^	Álvarez, *et al*.^12^	inf_dis_burden	4.062719
Bacterial disease cases (Derived determinant)	World Health Organisation Global Health Observatory^54^	Álvarez, *et al*.^12^	bac_dis	1.020674
*Climatic factors*				
Rainfall (mm) (Max)	World Bank^50^	This paper	rain_max	0.000074
Rainfall (mm) Average (Oct-Jan)	World Bank^50^	This paper	rain_oct_jan	0.000060
Rainfall (mm) Average (June-Sept)	World Bank^50^	This paper	rain_jun_sep_av	0.000036
Rainfall (mm) Average (Feb-May)	World Bank^50^	This paper	rain_feb_may_av	0.000018
Rainfall (mm) (Average of all months)	World Bank^50^	This paper	rain_tot_av	0.000008
Rainfall (mm) (Min)	World Bank^50^	This paper	rain_min	0.000004
Temperature [C] Average (Feb-May)	World Bank^50^	This paper	temp_feb_may_av	0.000003
Temperature [C] Average (Oct-Jan)	World Bank^50^	This paper	temp_oct_jan_av	0.000002
Temp [C] (Min)	World Bank^50^	This paper	temp_min	0.0000007
Temperature [C] (Average of all months)	World Bank^50^	This paper	temp_tot_av	0.0000003
Temperature [C] Average (June-Sept)	World Bank^50^	This paper	temp_jun_sep_av	0.0000002
Temp [C] (Max)	World Bank^50^	This paper	temp_max	0.0000002
*Surface transport Infrastructure*				
Surface transport infrastructure (Derived determinant)	Ministry of Road Transport and Highways Government of India^31^; Indian Railways, GoI^32^	This paper	road_rail_len	0.653450

**Table 2:** Human antibiotic consumption in India: Root mean square errors of prediction for training set using cross-validation for various component models.

	1 Component (*Population)*	2 Components *(Health infrastructure* + *Surface transport infrastructure)*	3 Components *(Total number of hospital beds* + *Surface transport infrastructure* + *Infectious disease cases)*
Cross-validation	583.2	376.0	492.2
Adjusted cross-validation	572.2	365.5	473.4

**Figure 2:**
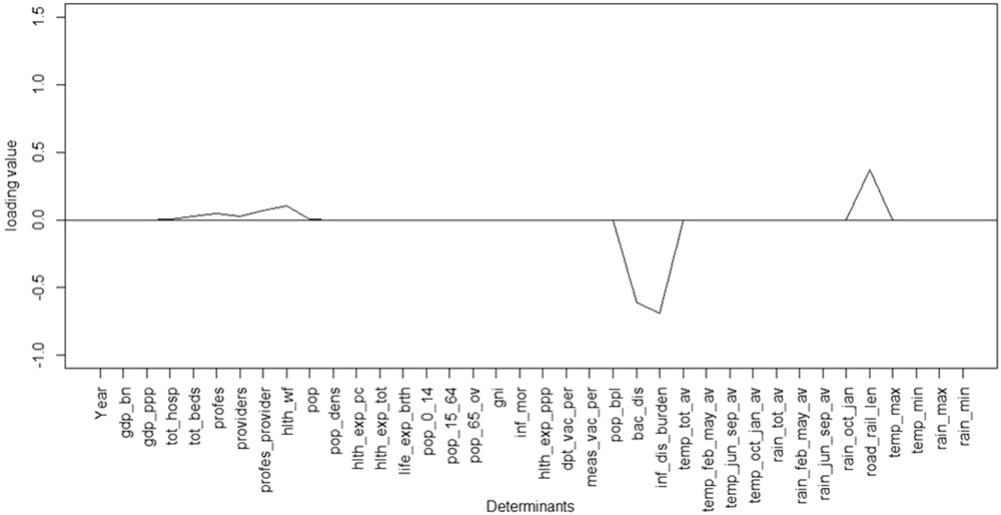
Human antibiotic consumption in India: Loadings value graph for determinants for 2 component model, loading values close to zero are not seen. For abbreviations see Table 1.

In case of several determinants the loading value^22^ was very close to zero (Fig. 2). For example, the Variable Importance Projection (VIP) score^22^ was 1.69 for the determinant ‘total population’ (Table 1), however, its loading value was very close to zero (Fig. 2), see methods section for explanation of VIP score and loading value. The VIP score for the derived determinants ‘Health infrastructure’ and ‘Surface transport infrastructure’ was 0.66 and 0.65 respectively (Table 1). Despite having low VIP score compared to the determinant total population (VIP score = 1.69) and the determinant infectious disease cases (VIP score = 4.06), the health infrastructure determinant displayed a higher loading value of 0.101 (Fig. 2) as compared to the value of total population and infectious disease cases (loading value = −0.69). Similarly, the loading value for surface transport infrastructure, 0.371 was also higher than the loading value for the total population and infectious disease cases determinants. Hence, the determinants included in the models were selected on the basis of VIP as well as loading value. When models were formulated combining various components, a 2 component model which was a combination of ‘Health Infrastructure’ (number of hospitals, number of hospital beds, number of practitioners and number of other allied health workers) and ‘Surface Transport Infrastructure’ (total road length + total railway route length-Km), termed ‘HISTI’ (HISTI = Health Infrastructure + Surface Transport Infrastructure) emerged as the better model with statistically most significant predictors for antibiotic consumption in India as compared to other models. The root mean square error of prediction (RMSEP) for training set for the HISTI model (Health Infrastructure + Surface Transport Infrastructure) was 376.0 and for test set 297.3, which was lowest compared to other models (adjusted R^2^ 0.94, p < 0.05).

The equation (Equation 1) for the HISTI model for estimating antibiotic consumption was

1 $${\rm{Y}}=-\,2639.6+{0.000257}^{\ast }{\rm{A}}+{0.00263}^{\ast }{\rm{B}}$$

where, Y = Predicted antibiotic consumption. A = Value of Health infrastructure. B = Value of Surface transport infrastructure.

The estimates for antibiotic consumption in India for the years 2011–2014 are presented in Table 3 (Figures used for years 2000–2010 are found in methods section, Data set). The estimated antibiotic consumption in 2014 (at the time of our analysis, data for determinants was available only up to 2014) based on the HISTI model was 11,597 (95% confidence interval-7545, 15650) standard units per 1000 population for India. Using this figure, the projected estimate of total antibiotic consumption for India came to 14.37 billion standard units for 2014. When an attempt was made to estimate antibiotic consumption for various constituent states of India by applying the HISTI model (Table 4), Maharashtra, Karnataka and Uttar Pradesh appeared to be the top three consumers of antibiotics in India.

**Table 3:** Estimated human antibiotic consumption for India for 2011 to 2014.

Year	Antibiotic consumption (standard units per 1000 population) (95% CI*) *CI = *Confidence interval*
	Prediction by the HISTI model (HISTI = Health Infrastructure + Surface Transport Infrastructure)
2011	10787 (9153, 12421)
2012	11308 (9432, 13184)
2013	10220 (9715, 10724)
2014	11597 (7545, 15650)

**Table 4:** Estimated human antibiotic consumption disaggregated for states of India for 2014.

	Prediction by Health Infrastructure + Surface Transport Infrastructure (HISTI) model	
	Antibiotic consumption (standard units per 1000population) - 2014	Total antibiotic consumption (standard units) - 2014
***States***		
India	11,597	14,367,372,539
Andhra Pradesh	861	74,877,940
Assam	391	12,401,460
Bihar	354	35,954,529
Chhattisgarh	169	4,267,595
Gujarat	584	35,814,601
Haryana	169	4,496,004
Himachal Pradesh	106	737,131
Jharkhand	86	2,831,105
Karnataka	979	59,950,623
Kerala	627	22,119,274
Madhya Pradesh	690	52,161,635
Maharashtra	989	115,874,336
Manipur	37	94,233
Meghalaya (Data not available on Railway route length)	25	67,312
Odisha	640	26,752,699
Punjab	282	8,063,789
Rajasthan	687	48,749,849
Tamil Nadu	768	52,742,474
Tripura	51	192,304
Uttar Pradesh	899	189,949,206
Uttarakhand	89	920,234
West Bengal	722	66,322,675
Union territory of Delhi (Data not available on number of health visitors and health supervisors)	167	3,356,864

## Discussion

This study investigated the determinants of human antibiotic consumption for India using PLS regression analysis. It is, to our knowledge, the first attempt to explore determinants of human antibiotic consumption at the country level for India, one of the highest consumers of antibiotics for humans in the world^4^. A two component model named ‘HISTI’ that includes the derived determinants, ‘Health Infrastructure’ and ‘Surface Transport Infrastructure’, had the least Root mean square error of prediction, and emerged as the best predictor for antibiotic consumption in India. For the analyses we used antibiotic sales data^4^ termed as consumption data. It might however be, an overestimation of actual consumption data, caused e.g. by patient non-compliance due to adverse effects of treatment or a recovery from symptoms (e.g. Cizman 2003)^23^. We have however in this paper used the term consumption as is commonly done when approximating sales data for consumption^4,23^.

Human antibiotic consumption in India increased by more than 40% between 2000 and 2010 and it was suggested that antibiotic consumption is likely to increase further with the progress of the Indian economy^7,24–26^.Our model also estimated an increasing trend until 2014, till which time data on involved determinants was available for analysis. The HISTI model includes health infrastructure and surface transport infrastructure as determinants for antibiotic consumption and one or both of these components are likely to increase further in the developing economy of India and therefore for some time to come the HISTI model may remain applicable in predicting antibiotic consumption in India. This may become important e.g. for planning of policies for health care, management of antibiotic use, management of antibiotic resistance and management of antibiotic residues in the environment. The model may also become useful in settings similar to India, where information regarding these determinants is available at the country/state/province/region level.

Various studies have previously evaluated determinants for antibiotic use. In Europe, in two early studies, cultural and economic factors were described to govern self-medication with antibiotics^27,28^. In a study analyzing determinants of out-patient antibiotic use in 17 European countries (2000–2005), the factors that appeared to be significant were population income, demographic structure, density of general practitioners and their remuneration method^29^. Data from 19 European countries from 1999–2007 were further assessed together with a number of factors describing countries in terms of agriculture, culture, climate, demography, disease burden, education, healthcare organization and socioeconomics^13^. In this analysis, relative humidity, healthcare expenditure proportion to gross domestic product, feelings of distrust, proportion of population aged 65 years and above and availability of treatment guidelines as well as a higher proportion of the population describing themselves as religious were found to be associated with higher total antibiotic use. Factors giving lower use of antibiotics were, restrictions on marketing activities towards prescribers, lower population density, lower number of antibiotics available in the market, educational attainment and higher degree of atheism^13^. A recent review of studies on factors influencing antibiotic use in Europe, identified factors such as cultural determinants, a range of patient-related factors like illness perception, health-seeking behaviour, previous experience, antibiotic awareness, drug perception, diagnosis labeling, work ethos, perception of practitioners, and practitioner-related factors such as management of respiratory tract infections, initial training, antibiotic awareness, legal issues and practice context^5^. A study from China showed that it was mainly the economic factors that determined antibiotic use in China^14^.

In our study, demographic factors such as total population, population density and age of the population, economic factors such as gross domestic product (GDP), gross national income (GNI), healthcare expenditure, poverty level, health related factors like life expectancy, infant mortality rate, vaccination rate, infectious disease burden and climatic factors such as temperature and rainfall, did not appear to have influence on antibiotic consumption in India as much as health infrastructure and surface transport infrastructure. This appears logical as health infrastructure-health systems^4,26^ facilitates antibiotic use; without it even if diseases in need of antibiotics are prevalent but if facilities are not there to give access to antibiotics, antibiotics will not be utilized. Further, without proper surface transport infrastructure outreach of antibiotics (access) and also of health personnel will be adversely affected. This is in line with a discussion paper by Mavalankar (2016)^30^ and must be understood in the context of India, a vast country where health care and educated health care providers are predominantly available in cities. For the large rural population, rail and road networks facilitate access to healthcare. For a continental country and a growing economy for a long time to come, until saturation is reached, these determinants- health and transport infrastructure- are likely to continue to influence antibiotic consumption in India.

Our analysis has, to the best of our knowledge, for the first time brought out health infrastructure and transport infrastructure as the determinants governing antibiotic consumption for a country. Both of these were earlier not considered for evaluation, the way we used them in our analysis, particularly transport infrastructure. In India, for transport infrastructure, we considered road and rail transport, as most of the (more than 95%) freight and passenger transportation occurs using road and railways^31,32^, however, in countries/regions, where waterways are also used in a major way for passenger and freight transport, they should also be included as a component of surface transport as a determinant in analysis.

Our study confirms that provider/prescriber induced demand commonly termed as supplier induced demand plays a role in determining antibiotic consumption, this is in line with the systematic review by Leonard *et al*.^33^ which suggests that there is an association between physician density and healthcare consumption. Further, Filippini *et al*.^10^ found that in Switzerland an increase in the number of physicians at cantonal level caused an increase in the cantonal per capita antibiotic sales. A study from the U.S. also reports that number of physicians and clinics per capita were drivers of antibiotic prescribing rate^34^, which is in line with our finding that health infrastructure is a determinant governing antibiotic consumption.

Although our analyses projects an increase in antibiotic consumption in India, from 10.7 units/person in 2010 to 11.6 units/person in 2014, compared to some countries in Europe, the use is still comparatively low, e.g. in 2010 France used 23.1 units/person^4^. Thus, there is every likelihood that antibiotic consumption per capita is likely to increase in India for some time to come^4,7,24,25^. As antibiotic use increases, the likelihood of quantitative and qualitative increase in antibiotic resistance in bacteria in India is also likely^26^. Further, increase in antibiotic consumption will also cause increase in antibiotic residues and resistance in the environment. It is important that countries such as India, areprepared to tackle the problems associated with increasing antibiotic use mentioned earlier and this is a point to be considered for the ‘National Action Plans’ being set up in all such countries following the GAP of 2015^3^.

Bu *et al*.^14^ state that besides being useful for estimating and forecasting antibiotic consumption, various exposure models using potential determinants of antibiotic consumption may become useful in estimating environmental residue concentrations of antibiotics in regions where environmental concentration are largely unmonitored. They further suggest designing of spatially resolved models, as antibiotic consumption data are generally available at country level, but determinants may be available at sub-national level also. As has been demonstrated by us by elaborating the antibiotic consumption for the constituent states of India (Table 4), our model has the potential to be useful for estimating antibiotic consumption also at sub-national level.

In our study, there was a possibility that the determinants could be correlated and might have similar predictive information. In that case, ordinary multiple linear regression would have shown high variability and would have become unstable if the correlation among determinants was high. Also, in our dataset, the number of determinants (independent variables) was greater than the number of observations for the dependent variable. In this case, too, ordinary least square regression in its usual form would have been unable to find a unique set of regression coefficients that minimize the sum of the squared errors. Hence, we built the prediction model by using Partial Least Squares (PLS) regression to avoid over-fitting and to remove highly correlated determinants. PLS regression is a method for constructing predictive models when the independent factors are many and highly collinear^21^. The main aim of PLS regression is to identify the components that explain more variance between the predictors and the response variables. Further, we used leave-one-out cross validation for building a robust model.

PLS regression finds components that simultaneously summarize variation of the determinants while being optimally correlated with the response variable. For a univariate response (in this case antibiotic consumption), each iteration of the algorithm used by PLS regression assesses the relationship between the determinants X (independent variables) and response Y (dependent variable). The predictor data are then orthogonally projected onto the direction (a vector of weights) to generate scores T and U (See online Supplementary material). The scores are then used to generate loadings P and Q, which measure the correlation of the score vector to the original determinants and response. At the end of each iteration, the determinants and the response are “deflated” by subtracting the current estimate of the determinants and response structure, respectively. The new deflated determinants and response information are then used to generate the next set of weights, scores, and loadings.

PLS regression is a method which is suitable either where there are many correlated independent variables or where the number of independent variables are much more than the number of observations for the dependent variable and has earlier been used in a variety of studies such as chemical, microbiological, metabolomics, pharmaceutical and health systems studies looking at patient safety and patient engagement^35–40^. Our study appears to be the first using PLS regression for predicting antibiotic consumption.

The strength of our study is that we have used several determinants per year and have used a method recommended for such data, PLS regression^41,42^. For all the determinants, we have used publicly available data, which makes our method and analysis potentially useful for others and other countries also for predicting their future antibiotic consumption, where similar datasets for determinants are available. Further, the suggested model/methodology might also become useful in estimating antibiotic consumption in situations where access to actual antibiotic consumption data is difficult to obtain or is not available.

Data for all the determinants for the years 2000–2010 was extracted from relevant publicly available reliable sources’ like the World Bank, the World Health Organization and the Government of India. There can always be limitations in relation to data collection and management techniques of such data, but those data are on the other hand the best or only reliable and valid data available. The methods of their collection are described in official publications of these renowned organizations.

A limitation of our study is that, as we used a data in which so called standard units are used, we could not convert the data to DDDs (Defined Daily Doses), prescription or packages as the data was only available to us at an aggregated level. We could thus not use the WHO recommended system of using ATC (Anatomic Therapeutic Chemical classification) and DDD for presenting drug utilization data^43^. The data we have used is reported to cover about 95% of sales^4^. This means that probably our predictions give figures for antibiotic consumption 5% lower than actual. Further, we also did not include any patient perceived factors like inappropriate treatment protocols, corruption etc., which in one questionnaire study was considered as having higher barriers than distance^44^. Besides human use, antibiotics are also used in animal therapy, prophylaxis and growth promotion, however we could not include that in our current study. It would have been useful to compare our predicted values of human antibiotic consumption with the actual national pharmaceutical sales data values to understand the veracity of our model. However, financial constraints did not allow us to purchase such data for the years 2011–2014 or for the Indian states.

### Conclusion and future research

A model combining health infrastructure and surface transport infrastructure, the HISTI model, was developed using PLS regression and R statistical platform for predicting human antibiotic consumption in India. As one or both these components are likely to increase further in the developing economy of India, for some time to come the HISTI model may have its utility in predicting human antibiotic consumption for India. India being a medium human development index country^19^ (HDI rank-131/188) and also a lower middle income country, the prediction model formulated in this study may also become useful in predicting antibiotic consumption in other low and lower middle income countries having circumstances similar to India, but without access to actual measurements of antibiotic consumption, particularly because many such countries could have data on their health and transport infrastructure.

We have only analyzed human consumption data in this paper, it will be interesting to find out determinants for animal consumption and also for total (human + animal) antibiotic consumption for India.

With new antibiotic policies including the national action plan^45^ and ‘Swatch Bharat’^46^ (clean India) campaign coming in to practice, it is possible that both optimization of antibiotic use is underway in India, and also infectious disease burden is coming down. Social initiatives like Indian Initiative for Management of Antibiotic Resistance^47^, Chennai declaration^48^, and Jaipur declaration^49^ might also contribute to optimization of antibiotic use by increasing awareness among the public as well as health care providers. Stricter enforcement of prescription only drugs, specifically antibiotics, and prescribing protocols and guidelines may also help in optimization of antibiotic use. Studies in which such factors are included should be undertaken in future.

## Materials and Methods

### Dataset

The determinants evaluated for governing human antibiotic use in India are shown in Table 1. A set of potential primary determinants were considered for evaluation based on available literature that explored the influence of various factors on antibiotic use^7–14^. In addition, several India specific potential determinants were added for evaluation. For example, in India besides allopathic (western) medical practitioners, there are also AYUSH (Ayurveda, Yoga, Unani, Siddha, Homeopathy) practitioners, who also prescribe antibiotics. Therefore, the practitioners, hospitals and healthcare based on AYUSH were also taken into account, i.e. in our analysis, the determinant Total number of hospitals was derived by summing up number of allopathic hospitals and number of AYUSH hospitals; Total number of hospital beds included number of beds in both allopathic and AYUSH based hospitals; Total number of professionals included allopathic medical practitioners, dental surgeons and registered practitioners under AYUSH systems; Total number of providers included the total number of general nursing midwives, auxiliary nursing midwives, health visitors and health supervisors; a combined determinant “Total number of professionals and providers” included the total number of professionals and the total number of providers. Some ‘Derived Determinants’ combining (summarizing) primary determinants within the same category (Table 1), were also created. For creating a derived determinant called ‘Health Infrastructure’ all entities in the health sector, total number of hospitals (allopathic and AYUSH system), total number of hospital beds (allopathic and AYUSH system), total number of health professionals (allopathic and AYUSH practitioners and dental surgeons) and total number of providers (general nursing midwives, auxiliary nursing midwives, health visitors and health supervisors) were combined (summed together). Likewise, total road length and total railway route length (Kms) in the country were combined to form a determinant named ‘Surface Transport Infrastructure’. Since, all these primary determinants within a category had the same unit, we summed up the primary determinants to obtain the derived determinant. Similarly, for the derived determinant ‘Bacterial disease cases’ total number of cases of leprosy, pertussis, diphtheria, tetanus, neonatal tetanus, cholera and tuberculosis were combined and for the derived determinant ‘Infectious disease cases’ bacterial disease cases, malaria cases, Japanese encephalitis cases and measles cases were added together. Data for all these determinants for the years 2000–2010 was extracted from relevant publicly available reliable sources (Table 1)^31,32,50–54^. Human antibiotic consumption data for India for the years 2000 to 2010 (expressed as standard units /1000 population; standard units = number of doses sold in the country; a dose being a pill, capsule, or ampoule) was sourced from Van Boekel *et al*.^4^. Antibiotic sales data available at this source was used as a proxy for human antibiotic consumption. According to these statistics the antibiotic consumption in India increased from 7,413 standard units per 1000 population in 2000 to 10,608 standard units per 1000 population in 2010. The consumption figures for intermediate years were (year-sales/consumption data) 2001-7,031, 2002-7,392, 2003-7,564, 2004-7,371, 2005-7,721, 2006-8,926, 2007-9,160, 2008-9,460, 2009-10,152 standard units per 1000 population. All the data were inputted in MS Excel for further analysis. In some instances, when data was missing for a particular year for a variable, the missing data was imputed using the forecast function of MS Excel in which the values of the determinant for previous years was used to predict the missing value.

At the time of analysis, for some states and union territories of India, data for more than one category of determinants was not available. These states and union territories were not included in the analysis presented in Table 4.

### Statistical analysis and Modeling the antibiotic consumption

In this paper we have used data available for relevant independent variables, to build a regression model that could predict antibiotic consumption in the future. For this we attempted to establish relationships between the studied independent variables (Table 1) and the dependent variable - antibiotic consumption using partial least square regression.

All the analysis was performed using the R statistical language platform^20^. The prediction model was built using PLS regression. A leave-one-out cross validation was used to build a robust model. The data was divided into training set (75% of the available data) and test set (remaining 25% of the data). The training set is a subset of the original data which is used to discover potentially predictive relationship, while the test set is the remaining part of the original data which is used to assess the strength and utility of a predictive relationship. Both the sets, training and test, were created using random sampling technique to minimize any bias.

A model was built on the training set and was validated on the test set. The inclusion of a determinant in the prediction model was decided on the basis of combined evaluation of variable importance in projection (VIP) score (Table 1) and loading value (Fig. 2) of the determinant in the model components^22^.

Variable importance in projection (VIP) scores reflect the relative importance of each X variable (independent variable) for each X variate in the prediction model. (For example, in the current study, population is an independent variable, while actual population in each year is a variate).VIP coefficients thus represent the importance of each X variable (independent variable) in fitting both the X- and Y-variates (variates of dependent variable- in our study, antibiotic consumption for each year), since the Y-variates (antibiotic consumption) are predicted from the X-variates (in our study- each year value of various determinants). VIP allows to classify the X-variables (independent variables in Table 1) according to their explanatory power for Y (antibiotic consumption). The loading values get generated by the algorithm used by the PLS regression and assess the relationship between the independent variables (determinants) and dependent variable (antibiotic consumption). The predictor data are then orthogonally projected to generate scores T and U, which are then used to generate loading values which measure the correlation of the score vector to the determinants and response (antibiotic consumption). (See online Supplementary material for explanatory calculations). As stated earlier, these two (VIP score and loading value) were used together to decide inclusion of a determinant in the prediction model.

We studied the correlation of potential determinants with the consumption of antibiotics based on the year-wise data available for India. A sequential approach was followed to develop a prediction model that could estimate the antibiotic consumption.

The developed regression model was applied to the 2011–2014 determinant data of India to estimate the antibiotic consumption in the country in these years. Since, data for some determinants was available only until the year 2014 at the time we conducted this analysis, we limited our estimates up to 2014. For estimating the antibiotic consumption for the various states of India, we used the average proportion of the determinant of that state with that of India.

The general underlying model of PLSR is explained (supplementary material Equation S1) and the R code is shown in online Supplementary material.

### Data availability

The datasets analysed during the current study are publicly available at sources mentioned in the paper.

## Electronic supplementary material

Supplementary information
