# Supplementary material for: Determinants of Antibiotic Consumption - Development of a Model using Partial Least Squares Regression based on Data from India
Source: Sci Rep. 2018 Apr 23;8:6421. doi: 10.1038/s41598-018-24883-1 (PMC5913309; doi:10.1038/s41598-018-24883-1)
Supplement: Supplementary file 1 — Supplementary information [file 41598_2018_24883_MOESM1_ESM.pdf]

Supplementary information for the paper

Title: Determinants of Antibiotic Consumption - Development of a Model using Partial Least Squares Regression based on Data from India

Authors: Ashok J. Tamhankar, Shreyasee S. Karnik, Cecilia Stålsby Lundborg

The general underlying model of PLS regression

$X = TP^T + E$ Equation S1

$Y = UQ^T + F$

where,

X = *n (rows) x m (columns)* matrix of determinants; ***Y*** = *n (rows) x p (columns)* matrix of responses (antibiotic consumption); ***T*** and ***U*** = *n (rows) x l(columns)*matrices that are, respectively, projections of ***X*** (the X score, component or factor matrix) and projections of ***Y*** (the Y scores); ***P*** and ***Q*** = *m (rows) x l (columns)* and *p (rows) x l (columns)* orthogonal loading matrices; matrices ***E*** and ***F*** are the error terms, assumed to be independent and identically distributed random normal variables. The decompositions of ***X*** and ***Y*** are made so as to maximise the covariance between ***T*** and ***U***.

R code for building the prediction model to estimate human antibiotic consumption in India

Note: Comments to explain each line of code are in italics starting with "#"

```
# Import the data for model building

ind.subset = read.csv(file.choose(), header=T)


# 75% of the sample size

set.seed(896)

smp_size<- floor(0.75 * nrow(ind.subset))


# set the seed to make your partition reproducible

set.seed(457)

train_ind<- sample(seq_len(nrow(ind.subset)), size = smp_size)


# Create training set

train.subset<- ind.subset[train_ind, ]


# Create test set

test.subset<- ind.subset[-train_ind, ]


# Call the library pls to run PLSR

library(pls)


# Regression command

mod.subset<- plsr(ab_use~., ncomp=6, data = train.subset, validation = "LOO")
```

```
summary(mod.subset)
```

# Command to plot the RMSEP graph

```
plot(RMSEP(mod.subset))
```

# 2 components plot with loading values

```
par(mar = c(10,4,2,2), mgp = c(7, 1, 0))

plot(mod.subset, plottype="loadings", comps = 2, ylim=c(-1,1.5), xlab="Determinants", ylab="",
xaxt="n")

axis(1, at=1:38, labels=prednames(mod.subset), las=3)

title(ylab = "loading value", mgp = c(2.5, 1, 0))

abline(h = 0)
```

# Predict the antibiotic consumption values for test set

```
predict(mod.subset, ncomp = 2, newdata = test.subset)
```

# Command to plot the predicted vs. measured value of the test data

```
par(mar = c(10,4,2,2), mgp = c(4, 1, 0))

predplot(mod.subset, ncomp=2, newdata = test.subset, asp=1, line=TRUE, xlab="Measured
value")

title(ylab = "Predicted value", mgp = c(2.5, 1, 0))
```
